# Exploring the role of sex in the association of late chronotype on cardiorespiratory fitness

**DOI:** 10.14814/phy2.15924

**Published:** 2024-01-31

**Authors:** J. Matthew Thomas, Philip A. Kern, Heather M. Bush, Sarah J. Robbins, W. Scott Black, Julie S. Pendergast, Jody L. Clasey

**Affiliations:** ^1^ Department of Kinesiology and Health Promotion University of Kentucky Lexington Kentucky USA; ^2^ Department of Biology University of Kentucky Lexington Kentucky USA; ^3^ Center for Clinical and Translational Science University of Kentucky Lexington Kentucky USA; ^4^ Sanders‐Brown Center on Aging University of Kentucky Lexington Kentucky USA; ^5^ The Department of Internal Medicine, Division of Endocrinology University of Kentucky Lexington Kentucky USA; ^6^ Barnstable Brown Diabetes Center University of Kentucky Lexington Kentucky USA; ^7^ Department of Biostatistics University of Kentucky Lexington Kentucky USA; ^8^ University Health Service University of Kentucky Lexington Kentucky USA; ^9^ Saha Cardiovascular Research Center University of Kentucky Lexington Kentucky USA

**Keywords:** circadian phase, dim light melatonin onset, graded exercise test, sex difference

## Abstract

Circadian rhythms differ between young adult males and females. For example, males tend to be later chronotypes, preferring later timing of sleep and activity, than females. Likewise, there are sex differences in body composition and cardiorespiratory fitness. Few studies have investigated the association between circadian rhythms, cardiorespiratory fitness, and body composition. We sought to determine whether chronotype and circadian phase were associated with cardiorespiratory fitness, body composition, and anthropometric measures in sedentary males and females. Fifty‐nine adults participated in the study. Circadian phase and chronotype were measured using dim light melatonin onset (DLMO) and the Morningness–Eveningness Questionnaire (MEQ) score. We used peak oxygen uptake (VO_2peak_) results from a maximal graded exercise test to assess cardiorespiratory fitness. Body composition, BMI, and circumferences were collected as markers of adiposity. We observed a sex difference in the association between DLMO and VO_2peak_. For males, a later DLMO was associated with a lower VO_2peak_. VO_2peak_ did not vary based on DLMO in females. Later circadian phase was also associated with increased body fat percentage, fat mass index, and abdominal circumference in males, but not females. Collectively, these results suggest that males who are later chronotypes may be at risk of obesity and low cardiorespiratory fitness.

## 1 INTRODUCTION

The circadian system generates 24‐h rhythms and synchronizes, or entrains, these internal biological processes to the 24‐h day. For example, the circadian system promotes wakefulness and activity during the day, and rest and inactivity at night. However, there is individual variability in the entrainment of circadian rhythms (Fischer et al., 2017; Roenneberg et al., 2003). Some people are classified as “early birds” because they prefer to wake early in the morning, while “night owls” prefer to be awake late into the night. This individual preference in the timing of sleep and activity, termed chronotype, varies systematically throughout the lifespan for males and females (Roenneberg et al., 2003). Adolescents and young adults typically have the latest chronotypes, while older adults display earlier chronotypes (Roenneberg et al., 2004).

Late chronotypes typically have delayed (i.e., later timing) sleep and physiological rhythms compared to earlier chronotypes. Despite their later internal timing, late chronotypes often have to wake up early on work or school days according to their social obligations. Thus, late chronotypes experience chronic misalignment between their internal circadian timing and the social environment (Roenneberg et al., 2003, 2012). It is well established that chronic circadian misalignment is associated with increased risk of obesity, cancer, Type 2 diabetes, cardiovascular disease, and the metabolic syndrome (Brum et al., 2015; Liu et al., 2018; Mota et al., 2021; Schernhammer et al., 2003; Sladek et al., 2023; Torquati et al., 2018).

Epidemiological studies found that late chronotype was associated with increased morbidity, cardiovascular disease, diabetes, and metabolic dysfunction. (Makarem et al., 2020; Merikanto et al., 2013; Yu et al., 2015). Late chronotype was also associated with increased risk of all‐cause mortality, compared to morning chronotypes (Didikoglu et al., 2019). Several studies have also investigated the relationship between chronotype and measures of obesity (Teixeira et al., 2022). However, these studies found conflicting results. Studies spanning adolescents to older adults found that evening chronotypes had greater body mass indexes (BMI; kg·m^−2^) (Arora & Taheri, 2015; Bloom et al., 2022; Teixeira et al., 2022), while other studies in similar age groups found no relationship between chronotype and BMI, waist circumference, or body fat (Kandeger et al., 2018, 2019; Lai & Say, 2013; Mota et al., 2016; Sato‐Mito et al., 2011; Teixeira et al., 2018). Moreover, few studies have investigated the association between chronotype and other body composition measures that account for the absolute and relative abundance of fat and fat‐free masses. In addition, little is known about the association between chronotype and cardiorespiratory fitness, which often contributes to clinical assessments of cardiovascular health (Holtermann et al., 2015). Poor cardiorespiratory fitness, measured as a low peak oxygen uptake (VO_2peak_) or decreased time‐on‐treadmill during a maximal graded exercise test, is a risk factor of cardiovascular and metabolic diseases (Carnethon et al., 2003).

Chronotype changes systematically across the lifespan in both males and females. Chronotype is early in young children, latest in adolescents, and then becomes earlier again in middle and older ages (Fischer et al., 2017). Large cross‐sectional studies have also identified sex differences in chronotype, with adult males being later chronotypes than females until about the age of 50 years (Roenneberg et al., 2004, 2007). However, very little is known regarding biological mechanisms contributing to sex differences in chronotype. Laboratory studies in highly controlled environments have found differences in circadian parameters between males and females. For example, one study found that females had a shorter circadian period than males (Duffy et al., 2011). However, sex differences in the relationship between chronotype and cardiometabolic health measures are understudied.

Chronotype can be estimated using surveys, such as the Morningness–Eveningness Questionnaire (MEQ), which uses multiple choice questions to classify an individual based on their preferred time of sleep and activity, and the Munich chronotype questionnaire (MCTQ), which queries actual sleep time on work/school and free days (Horne & Ostberg, 1976; Roenneberg et al., 2003). Both methods have been used in numerous clinical studies to investigate the association between human circadian rhythms and health outcomes (Teixeira et al., 2022). However, chronotype questionnaires provide no physiological information of the internal circadian clock. Dim light melatonin onset (DLMO) is a well‐established, reliable measure of the timing, or phase, of the internal circadian clock and is strongly correlated with mid‐sleep on free days corrected for sleep debt (MSFsc), a measure of chronotype from the MCTQ, and MEQ (Kantermann et al., 2015). However, DLMO is rarely employed in epidemiological or clinical studies because it requires a light‐controlled environment and is labor‐intensive (at least 6‐h sample collection in very dim light, ending at habitual bedtime) (Baron et al., 2017).

We previously measured DLMO and cardiorespiratory fitness in a cohort of sedentary males and females (Thomas et al., 2020). Because there is a sex difference in circadian phase in young adults, but no study has investigated whether there are sex differences in the association between circadian timing and cardiorespiratory fitness, we sought to explore this question using our prior dataset. The purpose of this study was to perform a post hoc analysis of our previous study to explore the role of sex in the association between DLMO and cardiorespiratory fitness, body composition, and anthropometric measures in sedentary adults. We studied a cohort of healthy, sedentary, and primarily young adults because identifying circadian factors that contribute to metabolic risk in this population may offer strategies for effective interventions at early stages of metabolic dysfunction.

## 2 METHODS

### 2.1 Participants

Data for this study were collected from participants recruited for a previously reported study (Thomas et al., 2020). Participants were 18–45 years old who self‐reported as healthy, with no known cardiovascular disease, hypertension, psychiatric or sleep conditions, and were medication‐free (except contraceptives). The study sample was overall a young adult cohort (ages 18–39 years) and there was one participant outside of the young adult range (female, 45 years old). Participants had not traveled across more than 1 time zone in the 4 weeks before beginning the study, were not primary caregivers for children <2 years old, and had not participated in night or rotating shift work in the previous year. At the time of the study, the participants reported that they did not participate in greater than 2 h of moderate‐vigorous structured exercise each week.

### 2.2 Anthropometric and body composition measurements

Height was determined using a wall‐mounted meter stick and body mass using a calibrated electronic scale (XL200, Escali Corp). Abdominal, waist, and hip circumferences were measured according to guidelines established by the Airlie Conference Proceedings (Lohman et al., 1988). Body fat percentage, fat mass, fat‐free mass, and mineral‐free lean mass were measured using total body dual‐energy x‐ray absorptiometry (DXA; GE Lunar iDXA bone densitometer; Lunar Software, Version 14.10; Lunar Inc) scans administered and analyzed by one trained investigator. A negative urine pregnancy test was required for all females immediately prior to DXA scanning. Fat mass index and fat‐free mass index were calculated as kg·m^−2^ (VanItallie et al., 1990).

### 2.3 Maximal graded exercise test

Maximal graded exercise tests (GXT_max_) were performed using an indirect calorimetry testing system (Vmax Encore, Vyaire Medical) with an integrated ECG (60 Hz sampling rate; Cardiosoft v6.51, GE Healthcare) and a treadmill ergometer.

Participants performed an incremental treadmill protocol, with 2‐min workload stages. GXT_max_ tests were performed in the morning or evening, depending on the randomized group allocation in our previously reported study (Thomas et al., 2020). The test began with a walking speed of 5.1 km·h^−1^ and 0% grade and progressed with a 0.6 km·h^−1^ increase in speed and 2% increase in grade with each subsequent stage. Verbal encouragement was given throughout the test and participants were encouraged to provide maximal exertion. Oxygen consumption (VO_2_) was measured breath by breath and averaged over 1‐min intervals. All participants achieved volitional fatigue and met at least one of the following criteria: respiratory exchange ratio ≥1.1 (determined by 1‐min averaging) and/or ≥ 85% of age‐predicted maximal heart rate (Tanaka et al., 2001). GXT_max_ data from two male participants were unavailable due to treadmill equipment failure. The highest 1‐min averaged VO_2_ value observed during the GXT was defined as VO_2peak_.

### 2.4 Circadian phase and chronotype

The primary measure of internal circadian timing was DLMO, which is a reliable, well‐established physiological measure of circadian clock timing (Lewy & Sack, 1989). DLMO was assessed using an 8‐h protocol in dim light (<10 lux at the eye level). Participants were instructed to refrain from alcohol, chocolate, bananas, and any form of caffeine on the day of the DLMO, due to their potential influences on melatonin levels (Pandi‐Perumal et al., 2007; Voultsios et al., 1997). Participants were also asked to abstain from nonsteroidal anti‐inflammatory drugs (NSAIDs) for the duration of the study due to the potential influence on melatonin levels (Murphy et al., 1996). The DLMO procedure began 8 h before habitual sleep onset on free/weekend days, calculated using the MCTQ. Saliva samples were collected each hour, except during a 2‐h window, beginning 4 h before habitual bedtime, when samples were collected at 30‐min intervals. Saliva melatonin levels were measured by Solidphase Inc. (Portland, ME) using a BUHLMANN direct saliva radio immunoassay test kit (RK‐DSM2‐U, Schonenbuch, Switzerland). Participants were allowed to eat snacks containing no ingredients which alter melatonin levels, except during 30‐min prior to a sample collection. Participants were instructed to rinse their mouth with water 30 min and 15 min prior to sample collection. DLMO was defined (using linear interpolation) as the time when the saliva melatonin level exceeded and remained above 4 pg·mL^−1^. DLMO could not be calculated in two participants (one male and one female) due to an erratic melatonin profile and melatonin levels, which did not exceed threshold.

We also collected MEQ as a self‐report measure of circadian rhythms based on individual preference for daily timing of sleep and activities. The output of the MEQ is a composite score, which is used to categorize a participant as an early, intermediate, or late chronotype. MEQ score could not be calculated from two participants (two females) due to erroneous completion of the questionnaire. We also collected the MCTQ, however, mid‐sleep on free days (corrected for sleep debt; MSFsc) could not be reported in 19 of our participants due to alarm clock use on free days (Kantermann et al., 2015; Roenneberg et al., 2012). Thus, we did not include MSFsc in our study analyses.

### 2.5 Data analysis

The sample size for the current study was based on a power analysis conducted for a previously reported study, which was designed to detect a circadian phase shift and not sex differences (Thomas et al., 2020). Data were analyzed using SPSS (Version 28). Descriptive data are presented as mean ± SD. Data summaries were created for the overall dataset and for each sex and differences were assessed with an independent samples *t*‐test and Levene's test for equal variances. We conducted a Pearson correlation analysis to assess the association between DLMO and VO_2peak_ in the overall cohort, and when stratified based on sex. Next, we conducted a two‐way ANOVA analysis with an interaction term for circadian phase, analyzed as a continuous variable, and sex to determine the effect of DLMO on VO_2peak_. Because this was an exploratory study, we report effect sizes from a partial eta‐squared. Because MEQ is a subjective estimate of internal timing, we also investigated the association of MEQ and VO_2peak_ for the overall dataset and separately for males and females. To further explore the relationship between circadian parameters, VO_2peak_, and body composition, we performed a bivariate Pearson correlation analysis between circadian parameters and VO_2peak_ and body composition for the overall dataset and separately for males and females. *p* ≤ 0.05 was considered significant for analyses.

## 3 RESULTS

We recruited sedentary adult males (*n* = 19) and females (*n* = 40; Table 1). The cohort consisted of 39 participants who identified as White, 9 as Asian, 4 as Black, 6 as other/mixed race, and 1 as Hispanic. We found no differences in DLMO or MEQ between males and females (*p* = 0.223 and *p* = 0.557, respectively).

**TABLE 1:** Descriptive characteristics of participants.

Variable	Male (*n*)	Female (*n*)	Overall	*p*‐value*
Age (years)	22.9 ± 4.4 (19)	24.9 ± 6.1 (40)	24.3 ± 5.7 (59)	0.23
BMI (kg·m^−2^)	24.5 ± 2.7 (19)	24.3 ± 4.7 (40)	24.4 ± 4.2 (59)	0.80
Circadian measures				
MEQ	50.4 ± 8.8 (19)	48.7 ± 10.8 (38)	49.3 ± 10.1 (57)	0.56
DLMO (decimal time)	22.6 ± 1.6 (18)	22.0 ± 1.5 (39)	22.2 ± 1.5 (57)	0.22
Cardiorespiratory fitness				
VO_2peak_ (ml/kg/min)	44.9 ± 6.1 (17)	33.1 ± 5.9 (40)	36.6 ± 8.1 (57)	<0.01
Body composition				
Body fat percentage	26.7 ± 7.1 (19)	34.0 ± 8.4 (40)	31.6 ± 8.6 (59)	<0.01
Fat mass (kg)	19.6 ± 6.9 (19)	22.4 ± 9.6 (40)	21.5 ± 8.9 (59)	0.26
Fat‐free mass (kg)	52.3 ± 5.2 (19)	41.4 ± 5.9 (40)	44.9 ± 7.6 (59)	<0.01
Mineral‐free lean mass (kg)	49.7 ± 5.0 (19)	39.1 ± 5.5 (40)	42.5 ± 7.3 (59)	<0.01
Fat mass index (kg·m^−2^)	6.5 ± 2.2 (19)	8.4 ± 3.6 (40)	7.8 ± 3.3 (59)	0.02
Fat‐free mass index (kg·m^−2^)	17.4 ± 1.4 (19)	15.4 ± 1.9 (40)	16.0 ± 2.0 (59)	<0.01
Anthropometric				
Abdominal circumference (cm)	86.8 ± 8.5 (19)	83.2 ± 11.6 (40)	84.4 ± 10.7 (59)	0.24
Waist circumference (cm)	82.4 ± 8.7 (19)	74.4 ± 10.0 (40)	77.0 ± 10.2 (59)	<0.01
Hip circumference (cm)	99.5 ± 6.6 (19)	100.5 ± 9.6 (40)	100.2 ± 8.7 (59)	0.70

*Note*: Data are mean ± SD.

* *p*‐value from independent samples *t*‐test.

**FIGURE 1:**
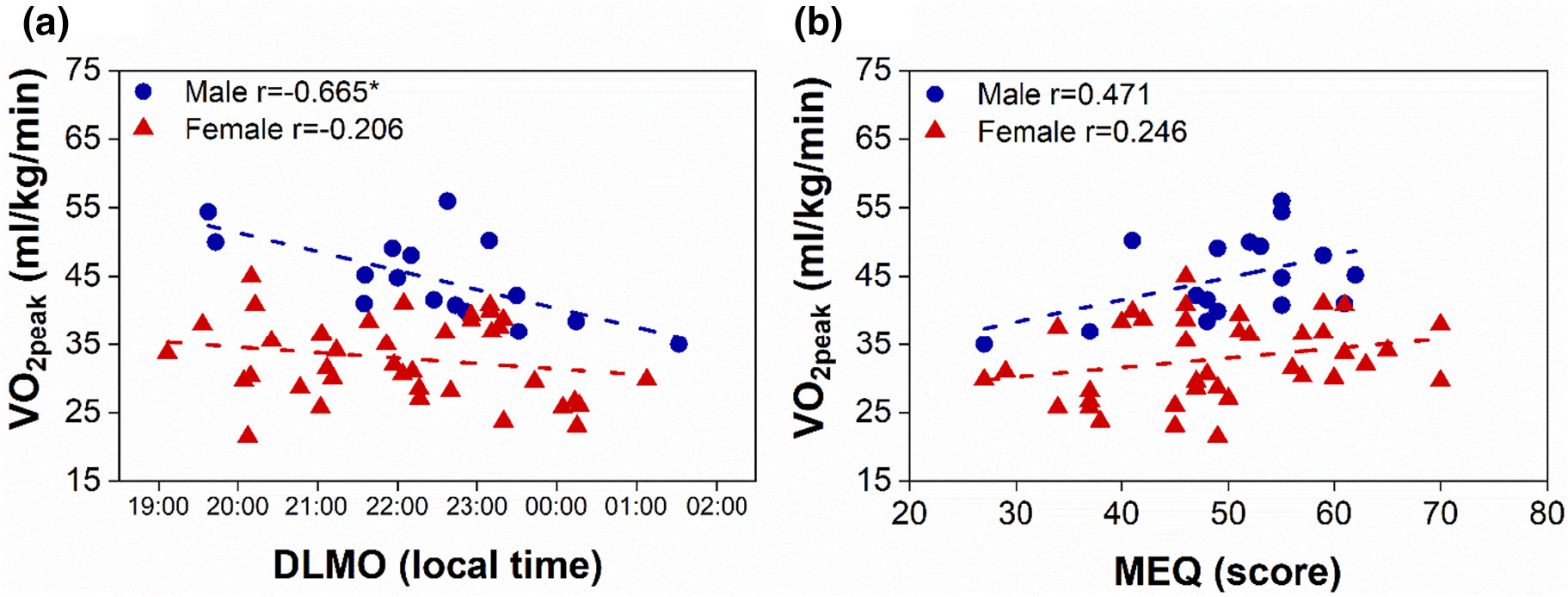
Later chronotype is associated with poorer cardiorespiratory fitness in males, but not females, in a sex‐stratified analysis. Correlation of DLMO (male *p* = 0.005*; female *p* = 0.209) (a) and MEQ (male *p* = 0.056; female *p* = 0.136) (b) with VO_2peak_ stratified by sex. Males are shown in blue circles and females are shown in red triangles. Dotted lines are linear fit. Pearson correlation *p < 0.05.

We first investigated the association between DLMO and VO_2peak_ (Figure 1a, data from all participants are shown). Although there was no association between DLMO and VO_2peak_ with the entire cohort (Table 2), the association of DLMO with VO_2peak_ differed depending on sex (Table 2; Table 3, two‐way ANOVA sex*DLMO interaction *p* = 0.090). The effect size, calculated as partial eta squared (*η*_*p*_^2^), was 0.06, indicating a medium effect (Cohen & Ebl., 1988). In males, but not females, a later DLMO was significantly associated with a lower VO_2peak_ (Figure 1a, Table 2). Next, we calculated predicted VO_2peak_ based on categorized DLMO values using the overall dataset (early DLMO = 5th percentile, intermediate DLMO = 50th percentile, and late DLMO = 95th percentile), and VO_2peak_ varied greatly by DLMO in males, whereas VO_2peak_ remained consistent among the range of DLMOs in females (Table 3). Based on the two‐way ANOVA model, VO_2peak_ ranged from 52.8 mL/kg/min in males with early DLMOs to 37.6 mL/kg/min in males with late DLMOs. In contrast, VO_2peak_ ranged only from 34.9 to 30.5 in females with early and late DLMOs, respectively. The VO_2peak_ achievement criteria used in our analysis was less rigorous than those used in lean, active individuals (Edvardsen et al., 2014; Mier et al., 2012). Therefore, we also analyzed our data using stricter VO_2peak_ criteria and our findings remained the same (Table S1). These data suggest that later internal clock timing was associated with worse cardiorespiratory fitness in males, but not females.

**TABLE 2:** Sex‐stratified Pearson correlation coefficients of DLMO, MEQ, body composition, and cardiorespiratory fitness.

Variable	DLMO			MEQ		
	Male^A^	Female	Overall	Male	Female	Overall
Cardiorespiratory fitness						
VO_2peak_	−0.67*	−0.21	−0.17	0.47	0.25	0.27*
Body composition						
Body fat percentage	0.49*	0.18	0.18	−0.27	−0.20	−0.23
Fat mass	0.46	0.06	0.13	−0.22	−0.14	−0.17
Fat‐free mass	−0.16	−0.18	−0.02	0.33	0.11	0.18
Mineral‐free lean mass	−0.15	−0.17	−0.01	0.31	0.06	0.15
Fat mass index	0.47*	0.10	0.13	−0.22	−0.20	−0.21
Fat‐free mass index	−0.24	−0.13	−0.06	0.48*	−0.03	0.12
Anthropometric measures						
Abdominal circumference	0.51*	0.03	0.17	−0.19	−0.15	−0.14
Waist circumference	0.21	−0.03	0.10	0.03	−0.12	−0.05
Hip circumference	0.21	0.01	0.06	0.02	−0.05	−0.04
BMI	0.17	0.07	0.10	0.12	−0.18	−0.12

^A^ Table values represent Pearson correlation coefficients; Pearson correlation *p < 0.05.

**TABLE 3:** Two‐way ANOVA analyses of DLMO and cardiorespiratory fitness.

	Predicted VO_2peak_ (95% CI)
Males	
Early DLMO (19.5)	52.8 (46.4–59.1)
Intermediate DLMO (22.0)	45.8 (42.9–48.8)
Late DLMO (25.0)	37.6 (31.9–43.2)
Females	
Early DLMO (19.5)	34.9 (31.4–38.5)
Intermediate DLMO (22.0)	32.9 (31.1–34.7)
Late DLMO (25.0)	30.5 (26.5–34.5)

*Note*: Table 3 represents predicted cardiorespiratory fitness values (VO_2peak_) using a regression model with different slopes for males and females. DLMO values were included in the model as a continuous variable and results are presented with representative (preselected) values for DLMO within the range of early (19.5), mid (22.0), and late (25.0). The slope estimate for males was −2.76 and the slope estimate for females was −0.81. Estimates of predicted VO_2peak_ for these representative values are presented by sex with 95% confidence intervals.

We also performed an exploratory analysis of the relationship between MEQ and VO_2peak_ (Figure 1b). Consistent with prior studies, DLMO was negatively correlated with MEQ (*p* < 0.001, Figure S1). MEQ was associated with VO_2peak_ in the entire cohort (*p* = 0.050; Table 2). However, when we stratified the analysis by sex, we found that the association between MEQ and VO_2peak_ was not significant in males (*p* = 0.056) or females (*p* = 0.136, Figure 1b).

**FIGURE 2:**
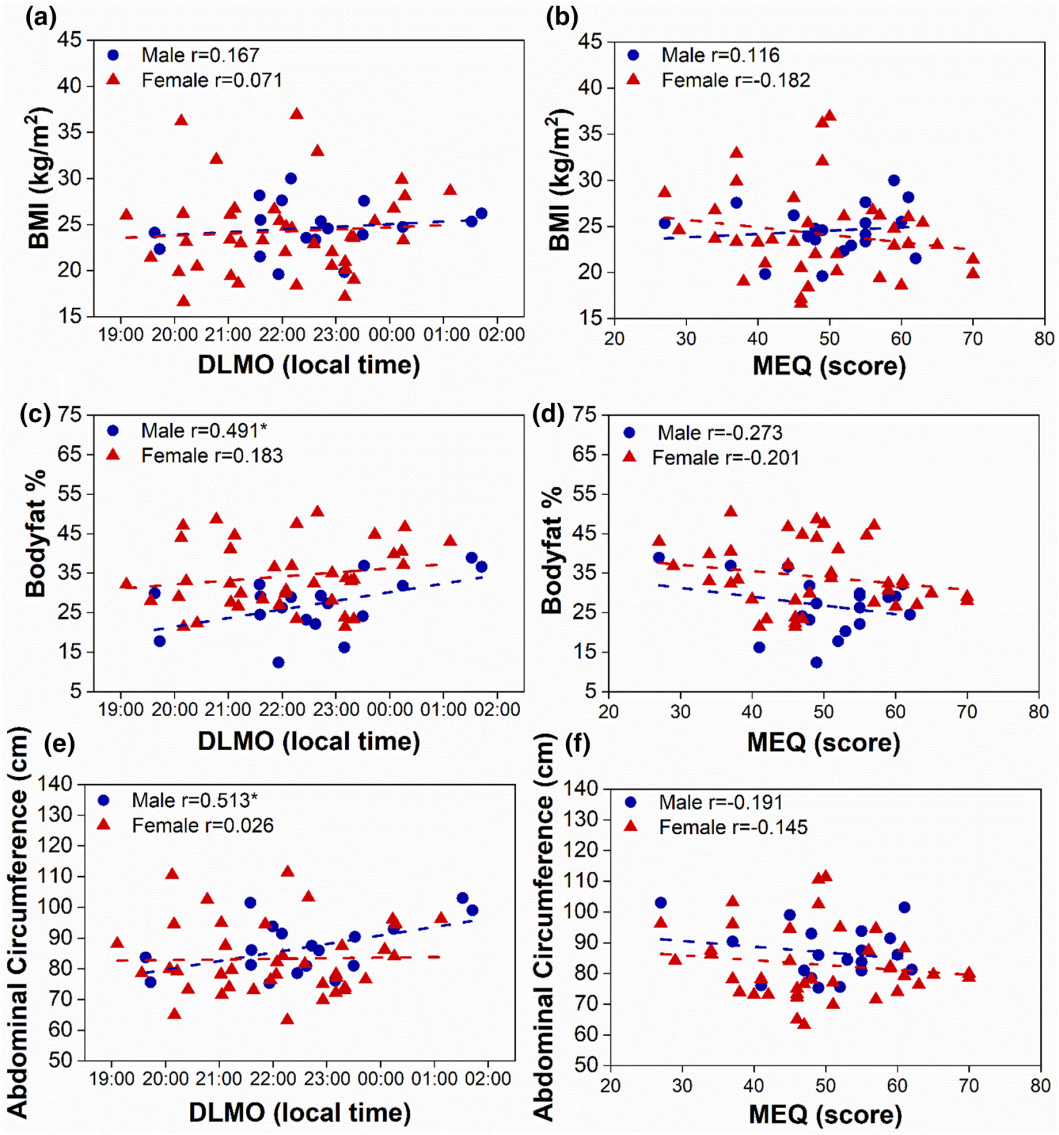
Later DLMO is associated with greater abdominal circumference and body fat percentage in males, but not in females. Correlation of DLMO (a,c,e) and MEQ (b,d,f) with BMI (a, male *p =* 0.508, female *p =* 0.666, b, male *p =* 0.636, female *p =* 0.274), body fat percentage (c, male *p =* 0.038*, female *p =* 0.265 d, male *p* = 0.258, female *p =* 0.226), and abdominal circumference (e, male *p =* 0.030*, female *p =* 0.877, f, male *p =* 0.432, female *p =* 0.384) stratified by sex. Males are shown in blue circles and females are shown in red triangles. Dotted lines are linear fit. Pearson correlation *p < 0.05.

**FIGURE 3:**
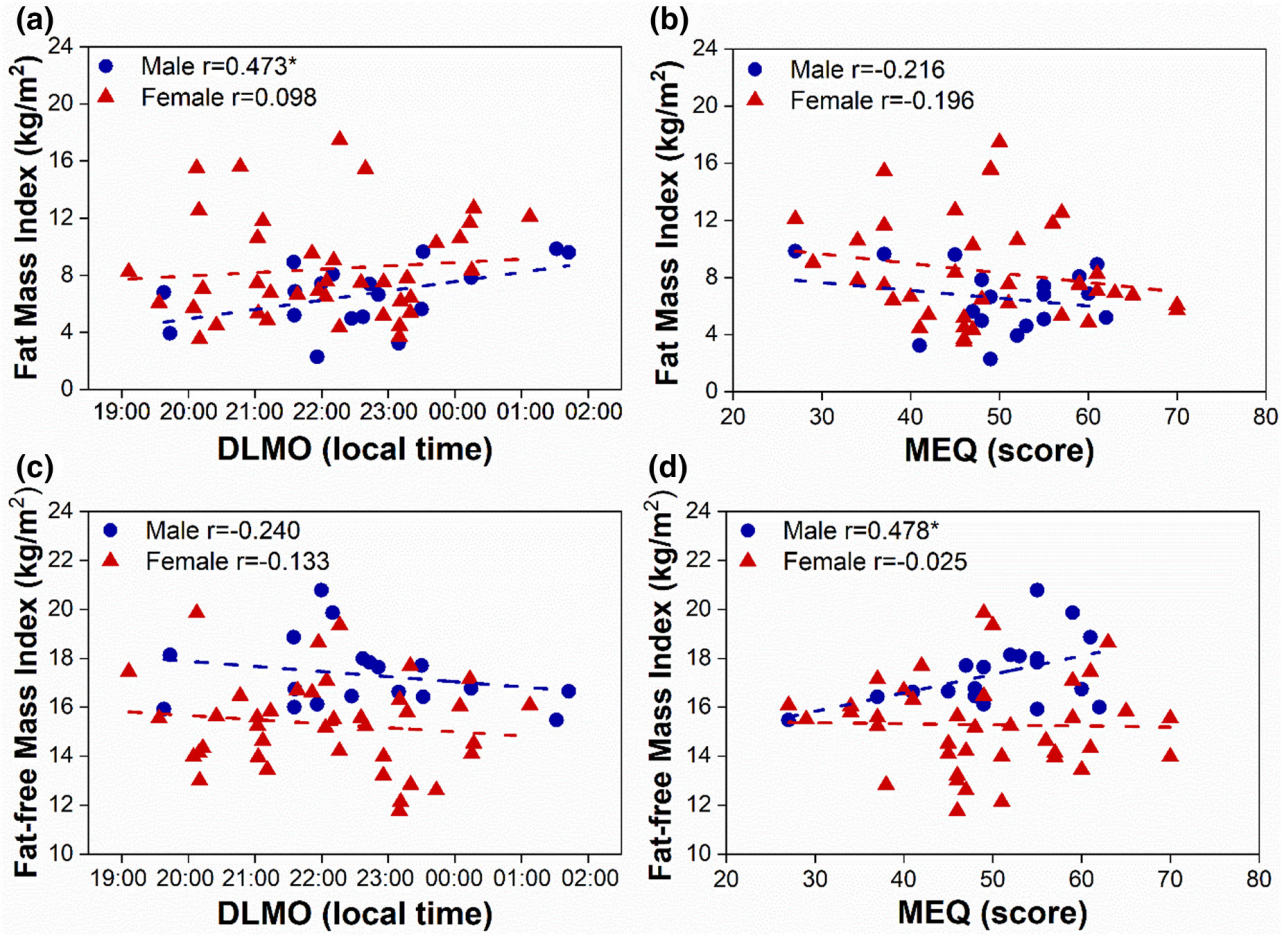
Later chronotype is associated with greater fat mass index and lower fat‐free mass index in males, but not in females. Correlation of DLMO (a,c) and MEQ (b,d) with Fat mass index (a, male *p =* 0.048*, female *p =* 0.554, b, male *p =* 0.374, female *p =* 0.238) and Fat‐free mass index (c, male *p =* 0.338, female *p =* 0.420 d, male *p* = 0.038*, female *p =* 0.879), stratified by sex. Males are shown in blue circles and females are shown in red triangles. Dotted lines are linear fit. Pearson correlation *p < 0.05.

Next, we investigated the relationship between circadian rhythms (DLMO and MEQ), body composition, and anthropometric measures. DLMO and MEQ were not significantly associated with any body composition or anthropometric measures when examining the entire cohort (Table 2). However, we further explored the relationship between circadian rhythms (DLMO and MEQ) and body composition measures by stratifying the data by sex and found that DLMO was significantly associated with abdominal circumference (*p* = 0.030 Figure 2e), body fat percentage (*p* = 0.038, Figure 2c), and fat mass index (*p =* 0.048, Figure 3a) in males, but not in females (*p* = 0.877, *p* = 0.265, and *p =* 0.554, respectively; Table 2). In addition, MEQ was significantly associated with fat‐free mass index in males (*p =* 0.038, Figure 3d; Table 2), but not in females (*p =* 0.879).

## 4 DISCUSSION

Circadian misalignment is pervasive in modern society (Roenneberg et al., 2012). Laboratory and observational studies have established that circadian misalignment is associated with adverse health consequences, including obesity and metabolic dysfunction (Baron et al., 2018; Brum et al., 2015; Scheer et al., 2009). Late chronotypes often have increased circadian misalignment due to a mismatch between internal rhythms and social obligations. In addition, late chronotype is associated with poor health behaviors, including smoking, increased consumption of alcohol and fast food, and decreased consumption of fruits and vegetables (Arora & Taheri, 2015; Sato‐Mito et al., 2011). Accumulating epidemiological evidence suggests that late chronotypes have poorer cardiometabolic health compared to intermediate or early chronotypes (Makarem et al., 2020; Merikanto et al., 2013; Yu et al., 2015). However, very little is known regarding the relationship between chronotype and cardiorespiratory fitness, which is often used as a clinical assessment tool for risk of cardiovascular disease and diabetes (Holtermann et al., 2015; Sawada et al., 2003). In this study, we found that the association between cardiorespiratory fitness and circadian phase depended on sex in apparently healthy adults. The results of the GXT showed that 8 males and 21 females in our study had a VO_2peak_ categorized as poor or very poor according to cardiorespiratory fitness classifications by American College of Sports Medicine (ACSM; [Liguori, 2022]). In addition, 5 males and 10 females had a VO_2peak_ categorized as fair, and 4 males and 9 females had a VO_2peak_ categorized as good or excellent. Thus, even though our participants were apparently healthy and not taking medications, our study included individuals with a range of cardiorespiratory fitness levels. We found that males who had a later circadian phase were more likely to have poorer cardiorespiratory fitness than males who had an earlier circadian phase. There was no significant association between circadian phase and cardiorespiratory fitness in females.

Other studies have found associations between chronotype, obesity, and measures of metabolic risk while adjusting for sex within a statistical model (Aguilar‐Galarza et al., 2021; Anothaisintawee et al., 2017; De Amicis et al., 2020). However, few studies have examined males and females separately, or investigated the interaction between sex and chronotype like we did in our study (Baron et al., 2017; Fárková et al., 2021; Yu et al., 2015). More studies focused on sex differences will be critical for understanding how the association between chronotype and sex changes across the lifespan. For example, some studies have found an association between chronotype, obesity, and metabolic risk in middle‐aged females, but not males, which is in contrast to our study that found that late chronotype is associated with metabolic risk factors in young males, but not females (Fárková et al., 2021; Maukonen et al., 2019; Yu et al., 2015). Chronotype changes throughout the lifespan as well as sex differences in chronotype (Fischer et al., 2017). Young adult males are typically later chronotypes than females but with age the trend reverses and females become later chronotypes than males (Fischer et al., 2017).

Our study also found that circadian phase and chronotype were associated with body composition, fat mass index, fat‐free mass index, and anthropometric measures in males, but not females. Abdominal visceral fat, estimated by abdominal circumference, is a risk factor of the metabolic syndrome (MetS). However, only 1 male and 11 female participants had abdominal circumferences that were above the cutoff for metabolic risk (NCEP ATP III MetS criteria (Expert Panel on Detection E, and Treatment of High Blood Cholesterol in A, 2001). In contrast, 16 male and 34 female participants had a body fat percentage categorized as poor or very poor according to ACSM guidelines (Liguori, 2022). Therefore, although only a subset of participants had abdominal circumferences associated with the MetS, most participants had excess body fat percentage, which increases the risk for cardiovascular and metabolic disease (Jo & Mainous 3rd., 2018). It is possible that a lower fat‐free mass index could also contribute to higher body fat percentage in our cohort. In fact, later chronotype in males was associated with increased body fat percentage as well as a decreased fat‐free mass index. Therefore, our findings support previous reported findings suggesting that late chronotype plays a role in exacerbating of obesity and is associated with overall body composition (De Amicis et al., 2020; Yu et al., 2015).

Late chronotype may be associated with increased obesity and metabolic risk for several reasons, including unhealthy behaviors and chronic circadian misalignment (Aguilar‐Galarza et al., 2021). However, potential mechanisms for the observed sex differences are unclear. We found no differences in circadian phase or MEQ between males and females in our study, so we cannot attribute the sex difference in the association between chronotype and metabolic risk simply to a sex difference in circadian timing. Although mechanisms are unknown, laboratory studies in highly controlled conditions found sex differences in circadian parameters (Cain et al., 2010; Duffy et al., 2011). For example, one laboratory study found that males had a shorter interval between DLMO and sleep onset, compared to females, which may result in circadian misalignment between endogenous circadian phase and sleep–wake rhythms (Cain et al., 2010). Future studies should investigate unhealthy behaviors, such as night eating, lack of physical activity, and poor nutrition in young adult males and females which could contribute to sex differences in the association between chronotype and metabolic risk factors.

A strength of our study is that we used an objective, physiological marker of internal circadian phase, DLMO. While it is challenging to measure DLMO, the alternative method is to use chronotype questionnaires, which are subjective and provide no physiological information about the internal circadian system. To our knowledge, no study to date has examined the relationship between DLMO and cardiorespiratory fitness, and very few studies have investigated the relationship between DLMO and measures of metabolic risk (Baron et al., 2017, 2018; Wong et al., 2022).

Another strength of our approach was that we investigated the relationship between circadian timing and a variety of body composition measures, rather than relying solely on BMI. Obesity status is sometimes misclassified using BMI because the body weight component of the equation does not differentiate between fat and fat‐free masses (Wong et al., 2021). Individuals who are classified as normal weight according to BMI but have excess total body or regional body fat are at high risk of cardiovascular and metabolic disease (Jo & Mainous 3rd., 2018). Also, measures of fat‐free mass, such as the fat‐free mass index, provide information about the nutritional status of an individual and are associated with physical activity levels (VanItallie et al., 1990; Westerterp et al., 1992). In our study, we found that males who were later chronotypes had a lower fat‐free mass index, which may indicate a lower level of daily activity in these individuals. Furthermore, abdominal circumference, which is associated with visceral adipose tissue, is a risk factor for the metabolic syndrome (Pouliot et al., 1994). We found associations between circadian timing and obesity indices of metabolic risk in males, but not females. There were no significant associations between BMI and circadian timing or chronotype in our study, so we would have missed important associations between chronotype and metabolic risk if we had measured only BMI.

There were some limitations to our study. First, we had a relatively small sample size, which was powered for a previously reported study designed to detect a circadian phase shift (Thomas et al., 2020). However, the current study is a post hoc examination, motivated by current best practice (including NIH guidance) to examine sex differences, even if underpowered. Nonetheless, even with our small dataset, we observed sex differences in the association between circadian phase and cardiorespiratory fitness. DLMO was our primary measure of circadian timing because it is an objective, physiological marker of internal circadian timing. A larger sample size would be more appropriate to examine sex differences between MEQ and fitness because MEQ queries the subjective preferences of an individual. Second, the GXT_max_ tests were performed in the morning or evening, depending on the randomized group allocation in our previously reported study (Thomas et al., 2020). There could have been time‐of‐day effects on performance in the GXT_max_ tests. However, we believe this is unlikely and that time‐dependent effects would be small. Similar to previous studies, we found no difference in VO_2peak_ for GXTs performed in the morning (VO_2peak_ = 37.2 mL·kg^−1^·min^−1^) versus evening (35.9 mL·kg^−1^·min^−1^) (Deschenes et al., 1998; Wung Burgoon et al., 1992). Third, we used VO_2peak_ criteria in this study of sedentary adults that were less strict than those typically employed using published guidelines (Liguori, 2022; Mier et al., 2012). To address this limitation, we conducted our primary analysis (two‐way ANOVA sex*DLMO interaction) using stricter VO_2peak_ criteria and our findings remained the same. Finally, we did not measure daily activity levels in our participants. The large variability in cardiorespiratory fitness in our cohort may, in part, be due to differences in daily activity levels.

## 5 CONCLUSIONS

In summary, our study demonstrates that sex is important to consider when designing experiments and circadian interventions for metabolic risk. Furthermore, our study may provide potential resolution for prior studies, which have mixed findings when examining the relationship between chronotype and obesity measures, perhaps because they combined data from males and females (Kandeger et al., 2018, 2019). In addition, we show that males with later circadian phase have poorer cardiorespiratory fitness and increased metabolic risk. Thus, male late chronotypes may represent a group at elevated risk for cardiovascular and metabolic diseases.

## AUTHOR CONTRIBUTIONS

JMT, PAK, JLC, and JSP designed the experiments. JMT, WSB, and JLC performed the experiments. JMT, HMB, SJR, JLC, and JSP analyzed the data. JMT, JLC, and JSP wrote the manuscript. All authors provided comments and approved the manuscript.

## FUNDING INFORMATION

This study was supported by a Barnstable Brown Diabetes and Obesity Research Center Pilot Award, a National Institutes of Health (UL1TR001998) Center for Clinical and Translational Science Pilot Award, the National Center for Advancing Translational Sciences (NIH TL1TR001997), NIH award T32 AG078110, the University of Kentucky Pediatric Exercise Physiology Laboratory Endowment, the University of Kentucky Arvle and Ellen Turner Thacker Research Fund, and the University of Kentucky. JSP was supported by an NIH award R01DK124774.

## CONFLICT OF INTEREST STATEMENT

The authors declare no conflicts of interest, financial or otherwise.

## ETHICS STATEMENT

The protocol for this study was reviewed and approved by the University of Kentucky Institutional Review Board (16‐0789‐F6A). Each participant provided written informed consent prior to participation in research procedures.

## Supporting information


Figure S1.
Table S1.

## Data Availability

Data will be made available upon request.
