# Supplementary material for: Exploring the role of sex in the association of late chronotype on cardiorespiratory fitness
Source: Physiol Rep. 2024 Jan 31;12(3):e15924. doi: 10.14814/phy2.15924 (PMC10830391; doi:10.14814/phy2.15924)
Supplement: Supplementary file 1 — Figure S1. Table S1. [file PHY2-12-e15924-s001.docx]

**Supplemental Material**

**Exploring the role of sex in the association of late chronotype on cardiorespiratory fitness**

J. Matthew Thomas, Philip A. Kern, Heather M. Bush, Sarah J. Robbins, W. Scott Black, Julie S. Pendergast, Jody L. Clasey

|  |
| --- |
| **Supplemental Figure S1.** Dim light melatonin onset (DLMO), a physiological measure of internal circadian timing, was associated with morningness-eveningness questionnaire (MEQ) score. |

**Supplemental Table S1.**

|  | **Two-way ANOVA slope** | ***p*-value** |
| --- | --- | --- |
| Males | -2.83 | 0.059 |
| Females | -0.71 |  |

Two-way ANOVA analysis with an interaction term for circadian phase and sex. Achievement of VO_2peak_ for this analysis was defined as meeting a minimum of two of the following criteria: respiratory exchange ratio ≥1.1 (determined by 1-minute averaging), RPE ≥ 17, and/or age-predicted maximal heart rate achieved or exceeded. N=46
